# Antibacterial, Transparency, and Mechanical Properties of Cationic Radical Initiator Triggered Polystyrene Sheets Obtained by Thermal Blending

**DOI:** 10.3390/polym16223167

**Published:** 2024-11-13

**Authors:** Hiroki Maruyama, Akihiro Kishi, Yuki Konoeda, Hiroshi Ito, Toshikazu Tsuji

**Affiliations:** 1 KIRIN Central Research Institute, Kirin Holdings Co., Ltd., 26-1 Muraoka-Higashi 2-chome, Fujisawa 251-8555, Kanagawa, Japan; 2 Graduate School of Organic Materials Science, Yamagata University, 4-3-16 Jonan, Yonezawa 992-8510, Yamagata, Japanihiroshi@yz.yamagata-u.ac.jp (H.I.); 3 Research Center for GREEN Materials and Advanced Processing (GMAP), 4-3-16 Jonan, Yonezawa 992-8510, Yamagata, Japan

**Keywords:** polystyrene, antibacterial additives, thermal blending, cationic moiety

## Abstract

Polystyrene (PS) is widely used because of its transparency, mechanical strength, and ease of production. With rising health concerns, antibacterial PS is increasingly sought after, but few polymer-based antibacterial agents have been prepared to date. In this study, polystyrene was synthesized using a cationic radical initiator, 2,2′-azobis-[2-(1,3-dimethyl-4,5-dihydro-1*H*-imidazol-3-ium-2-yl)] propane triflate (ADIP), and evaluated as an antibacterial additive. The PS polymerized with ADIP (ADIP-PS) was prepared with number-average molecular weights (*M_n_*) from 15,000 to 40,000. Further, blending 5–10% ADIP-PS with an *M_n_* of 23,000 into general-purpose polystyrene (GPPS) provided antibacterial activity against *Staphylococcus aureus* while maintaining the transparency and strength of GPPS. Surface analysis revealed hydrophilic properties and exposed cationic groups, as confirmed by contact angle measurement and anionic dye titration, respectively. In addition, the antibacterial activity increased with higher cationic group concentrations, particularly at lower molecular weights. This method presents a promising approach to introducing antibacterial properties to PS products.

## 1. Introduction

Polystyrene (PS) is a widely used plastic because of its transparency, tensile strength, ease of production, and durability [1,2]. In particular, its transparency makes it ideal for food packaging, allowing for the visual inspection of food freshness, and its strength helps maintain shape and protect contents [3,4]. High-impact polystyrene is also used for household appliances, offering high impact resistance [5,6], whereas syndiotactic polystyrene is employed in automotive and electrical components because of its superior heat and chemical resistance [7,8]. Along with polyethylene, polypropylene, polyvinyl chloride, and polyethylene terephthalate, PS is one of the most produced synthetic resins [9]. Recently, efforts have been made to reduce the environmental impact of PS, including the development of bio-based polystyrene synthesized from styrene derived from *Escherichia coli* or yeast [10,11,12]. Overall, PS is integral to daily life, and its applications continue to expand.

However, as global health and safety concerns grow, demand for antibacterial functionality in polymer products has surged [13,14,15]. PS is in frequent contact with humans, making it an important polymer target [16]. Antibacterial properties can be imparted to PS through various methods, including blending or coating with antibacterial agents [17,18,19]. Although blending low-molecular-weight antibacterial agents into PS is common, concerns about their environmental impact and longevity persist [20,21]. In contrast, coating with contact-killing-type antibacterial polymers offers greater durability [22]. However, industrial coating processes often require multiple steps, increasing complexity. Blending polymer-based antibacterial agents into PS presents a more efficient industrial approach, but few studies have addressed this challenge.

**Figure 1:**
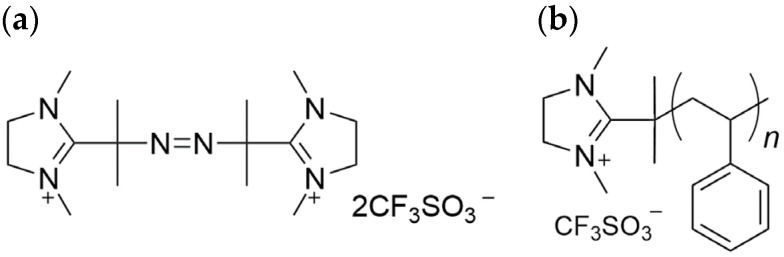
Chemical structures of the (**a**) initiator ADIP and (**b**) polymerized PS with ADIP attached to the ends of the PS chains.

In recent years, polymerization initiators that impart functionality to polymers have gained attention as an important research area. These initiators provide functions such as grafting onto surfaces and molecular orientation within polymer matrices, enabling unique applications like biomedical science. For example, studies have been conducted using cholesterol-based modifiers to form graft coatings with amino groups on glass surfaces to control the orientation of liquid crystals and utilizing halogen bonding between perfluoroalkyl iodides and amines to develop efficient synthesis methods for fluorine-containing polymers [23,24]. In a previous study, we demonstrated that thermally pressing polystyrene synthesized with the cationic radical polymerization initiator 2,2′-azobis-[2-(1,3-dimethyl-4,5-dihydro-1*H*-imidazol-3-ium-2-yl)] propane triflate (ADIP; Figure 1a) produces a resin with an antibacterial surface [25]. ADIP, an azo polymerization initiator with imidazolium-based quaternary ammonium groups, incorporates cationic groups at the polymer chain ends during polymerization, giving the material antibacterial properties [26]. In the case of ADIP-polymerized polystyrene (ADIP-PS; Figure 1b), these cationic groups align on the resin surface, resulting in contact-killing antibacterial activity [25]. However, ADIP-PS has drawbacks, such as reduced transparency and strength compared to PS synthesized using a general polymerization initiator.

In this study, we aim to balance the antibacterial properties of ADIP-PS with the transparency and mechanical strength of general-purpose polystyrene (GPPS) by blending ADIP-PS with commercial GPPS. To investigate the effect of the molecular weight on blending, we synthesized ADIP-PS with various molecular weights. The sample with the highest yield was selected, and the optimal blending ratio of ADIP-PS to GPPS was determined to maintain acceptable transparency and mechanical strength. Subsequently, we evaluated the effects of the ADIP-PS blending ratio on the surface properties and antibacterial activity of the PS blend. We also examined the relationship between these properties and the molecular weight of ADIP-PS.

## 2. Materials and Methods

### 2.1. Materials

Styrene was purchased from Tokyo Chemical Industry Corporation (Tokyo, Japan) and passed through an inhibitor removal column (Sigma-Aldrich, St. Louis, MO, USA) to eliminate *tert*-butylcatechol. Toluene, benzyl alcohol, and methanol were obtained from FUJIFILM Wako Pure Chemical Corporation (Osaka, Japan). The cationic radical initiator ADIP was synthesized according to a previously described method [26]. ADIP synthesis involved three steps, starting with the neutral radical initiator 2,2′-azobis(isobutyronitrile) (AIBN). First, the cyano groups of AIBN were converted into imidate groups under acidic conditions. *N*-Methylenediamine was then attached to the imidate groups, followed by cyclization to form imidazoline rings. Finally, methylation using methyl trifluoromethanesulfonate under mild conditions produced ADIP. Ultrapure water, prepared using a Milli-Q system (Q-POD1; LC-Pak Polisher, Millipore, Billerica, MA, USA), was used in all experiments. GPPS (MW1D, *M_n_* = 1.1 × 10^5^, *M_w_* = 3.5 × 10^5^) was sourced from Toyo Styrene Corporation (Tokyo, Japan).

### 2.2. Synthesis of ADIP-PS

For all ADIP-PS samples, styrene (150 mL) and toluene (50 mL) were added to a 300-mL separable flask and stirred at 100 rpm using a four-rotor spring mixing impeller. Samples AD-15K and AD-23K were polymerized at 40 °C, whereas AD-40K was polymerized at 60 °C. After reaching the set temperature, ADIP (8.73 g) was dissolved in benzyl alcohol (30.6 g) and added to the flask. ADIP was added at a concentration of 1 mol% relative to the styrene monomer. In a previous study, the initiator efficiency of ADIP at 20 °C was estimated to be 0.51, which is comparable to conventional azo-initiators [27,28,29]. The polymerization reactions for AD-15K and AD-40K proceeded for 24 h, whereas that of AD-23K required 48 h. The differences in the polymerization conditions for each ADIP-PS sample are listed in Table 1. After polymerization, the resulting polymer was precipitated by pouring the reaction solution into methanol. The liquid fraction was then decanted and the polymer precipitate was washed with methanol and dried under reduced pressure to yield a powder.

### 2.3. Characterization of Synthesized ADIP-PSs

The molecular weights of the ADIP-PS samples were determined using gel permeation chromatography (GPC) (HLC-8220GPC, Toso, Tokyo, Japan) with a Shodex GPC KF-405L HQ column (Showa Denko, Tokyo, Japan) at 40 °C. Calibration was carried out with polystyrene standards (Cat 81434-1EA; Sigma-Aldrich, St. Louis, MO, USA) using chloroform as the eluent. Attenuated total-reflection Fourier transform infrared spectroscopy (ATR-FTIR) was used to identify the functional groups in PS, and a Nicolet IS50 FTIR spectrophotometer (Thermo Fisher Scientific, Waltham, MA, USA) equipped with an ATR attachment, diamond crystal plate, and a deuterated triglycine sulfate detector was used. Spectra were measured between 500 and 4000 cm^−1^ and averaged over 64 scans. The nitrogen content (%, N content) was analyzed using a CHN coder MT-5 analyzer (Yanaco, Kyoto, Japan). Based on previous reports assuming that 1.8 initiator termini bind to PS [30,31], the ADIP residue content (mol%) was calculated from the nitrogen content using Equation (1).

(1) $$ADIP\;residue\;content\left(mol\%\right)=\frac{1.8\times M_{ADIP}}{M_{ADIP-PS}}$$

Here, *M_ADIP_* is the molecular weight of ADIP (144.2 g/mol), and *M_ADIP-PS_* is the molecular weight of ADIP-PS ((1.8 × 28)/N content).

### 2.4. Preparation of PS Sheets

Pure ADIP-PS sheets were pressed and molded from the synthesized ADIP-PS powder. The powder was first sandwiched between polyimide films (UPILEX-50S, UBE Corporation, Tokyo, Japan), which acted as release agents, and then placed between stainless steel (SUS) plates. The assembly was pressed at 140 °C for 20 s per press. The heat-pressed sheet was removed from the films, cut into four sheets, and sandwiched together again for ten additional press cycles. Polymer blends of ADIP-PS and GPPS were prepared using a 3S150 twin-screw melt-kneading extruder (Toyo Seiki Seisaku-Sho Ltd., Tokyo, Japan) at 190 °C with a screw rotation of 100 rpm. The polymer blends were ground into pellets, sandwiched between polyimide film and SUS, and pressed at 230 °C and 10 MPa for 8 min. The resulting round sheets had a diameter of 100 mm and a thickness of 0.1 mm and were used for antibacterial activity evaluation, contact angle measurements, and surface cationic group analysis. For transmission evaluations, 25-mm-diameter sheets of specific thickness were prepared using a SUS mold placed between polyimide films.

### 2.5. Characterization of PS Sheets

#### 2.5.1. Transmittance Evaluation

The transmittance of the PS sheets was measured over wavelengths from 380–1050 nm using an angle-dependent total reflection device (Lambda Vision Inc., Kanagawa, Japan) with a D65 light source.

#### 2.5.2. Tensile Tests

The tensile strength was measured following the ISO 527-2:2012 standard using a universal testing machine (Strograph VGS-E01; Toyo Seiki Seisaku-Sho Ltd., Tokyo, Japan) at a crosshead speed of 5 mm/min [32]. Test specimens molded into a dumbbell shape were used (ISO 527-2:2012 standard -5B), with a parallel section width of 2 mm, a parallel section thickness of 1 mm, and a specimen length of 35 mm. The chuck distance was 20 mm, and the strain was calculated based on the displacement relative to the initial chuck distance. The values for tensile strength and fracture strain were calculated from the obtained stress-strain curves. The Young’s modulus was calculated from the values of the stress-strain curve in the range of low stresses.

#### 2.5.3. Contact Angle Measurements

Contact angle measurements were taken using a contact angle meter (DMo-502, Kyowa Interface Science, Saitama, Japan) with purified water droplets (4 μL). Measurements were taken at six different locations on the sheet, and the average contact angle was calculated.

### 2.6. Antibacterial Activity Evaluation

Antibacterial activity was evaluated following the ISO 22196:2011 standard [33], which measures antibacterial activity on plastic surfaces against *Staphylococcus aureus* (NBRC12732). Polyethylene sheets, which do not have antibacterial properties, were used as controls. *S. aureus* was cultured for 24 h on standard agar medium (5 g/L meat extract, 10 g/L peptone, 5 g/L NaCl, and 15 g/L agar). Colonies were then suspended in 1/500-diluted nutrient broth to achieve a bacterial concentration of 2.5–10 × 10^5^ colony forming units (CFU). This solution was applied to the reference and test PS samples, which were incubated at 35 °C for 24 h in 90% humidity. Samples were washed with soybean–casein-digest–lecithin–polysorbate medium (10 mL), serially diluted (10×, 10^2^×, 10^3^×, and 10^4^×), and 1 mL of each dilution was plated on standard agar. After solidification, the agar plates were incubated at 35 °C for 40–48 h, and colonies were counted to calculate log (CFU/cm^2^). When the colony counts were zero, the log (CFU/cm^2^) was set to 0.05 for convenience.

### 2.7. Live/Dead Assay Method

A *S. aureus* suspension with a concentration of 1 × 10^9^ CFU/mL (0.1 mL) was dropped onto PS sheets and PS sheets kneaded with ADIP-PS (diameter of 4 cm). The sheet was incubated at 35 °C for 3 h under a humidity of at least 90%. The *S. aureus* was stained using a LIVE/DEAD BacLight Bacterial Viability Kit (Cat. No. L13152, Thermo Fisher Scientific). A working solution was prepared by diluting a mixture of SYTO 9 and propidium iodide (PI) 100-fold with water. Subsequently, 1 μL of the working solution was added to 9 μL of the bacterial suspension on the PS sheets and PS sheets kneaded with ADIP-PS, and the mixture was stained for 15 min. The SYTO 9 dye fluoresces green for live and dead bacteria, and PI fluoresces red to indicate cell-membrane-damaged bacteria. The stained samples were examined, and images were captured by a confocal microscope (LSM980, ZEISS, Jena, Germany) equipped with a Plan Apochromat 63× oil immersion objective with a 1.4 NA.

### 2.8. Confirmation of Cationic Groups on the Surface

To confirm the presence of cationic groups on ADIP-PS sheets, we measured the adsorption of the anionic fluorescent dye 6-(*p*-toluidino)-2-naphthalenesulfonic acid sodium salt (TNS) [25]. A 25 μM TNS solution was prepared by dissolving TNS in ethanol and diluting it tenfold with water. To detect the surface cationic groups, 2.5 μM TNS solution (40 μL) was applied to the ADIP-PS samples, covered with an 18 mm × 18 mm square glass, and left at room temperature (approximately 25 °C) for 1 min. Subsequently, the cover glass was removed, and the TNS solution (10 μL) was collected and mixed with water (90 μL) and dioxane (400 μL). The fluorescence intensity at 421 nm (excited at 370 nm) was measured using a fluorescence spectrophotometer (FP-8500, JASCO, Tokyo, Japan). The TNS concentration was determined from a calibration curve, and the adsorption percentage was calculated.

## 3. Results

### 3.1. Synthesis of ADIP-PS with Various Molecular Weights

**Figure 2:**
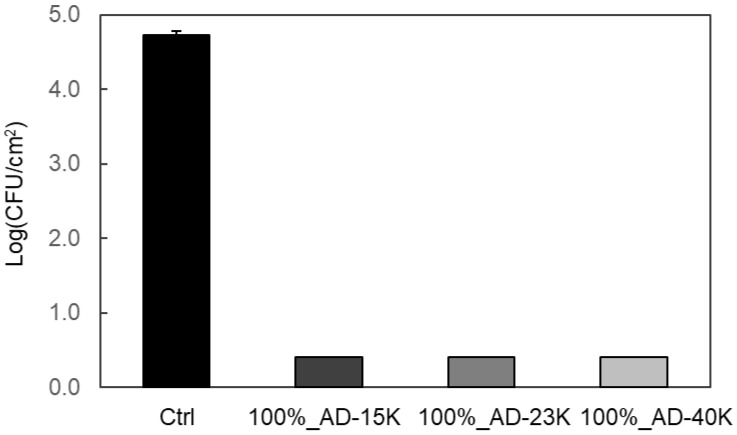
Antibacterial activity of 100%_ADIP-PS sheets against *S. aureus*. Error bars represent the standard deviation (SD) with *n* = 3.

ADIP-PS was synthesized as an antibacterial additive. Using toluene as the solvent at 40 °C for 24 h, ADIP-PS (AD-15K) with an *M_n_* of 15,000 and *M_w_* of 54,000 was obtained in 40% yield. Because of the relatively low molecular weight of AD-15K, we attempted to synthesize higher-molecular-weight ADIP-PS by increasing the reaction temperature or extending the reaction time. ADIP-PS (AD-23K), synthesized over 48 h, had a *M_w_* of 120,000 and a 56% yield, whereas ADIP-PS (AD-40K), synthesized at 60 °C, had a *M_w_* of 310,000 and a 33% yield (Table 2). The ADIP content in PS was calculated by determining the number of nitrogen atoms exclusively in ADIP, revealing the proportion of ADIP relative to the entire molecule decreases as *M_w_* increases (Table 2). FTIR analysis confirmed the absence of residual styrene, indicated by the disappearance of the band at 1683 cm^−1^, which corresponds to the double-bond stretching vibration of the vinyl group. The presence of ADIP functional groups was confirmed by bands at 1150 and 1270 cm^−1^, characteristic of trifluoromethanesulfonate (triflate) ions, likely corresponding to CF_3_ and SO_3_ stretching modes (Figure S1). Additionally, strong bands at approximately 750 and 700 cm^−1^, associated with out-of-plane CH bending in the benzene ring, were observed, and these are characteristic of PS. Based on these findings, the synthesis of ADIP-PS was confirmed. Notably, when AD-23K, a representative ADIP-PS, was left at 90 °C for 72 h and analyzed by FTIR, no significant changes were observed in the bands derived from the functional groups of ADIP (Figure S2). This suggests that ADIP is stably bonded with the PS. Given that ADIP-PS with a *M_w_* similar to GPPS was obtained, further investigation proceeded using these three ADIP-PS samples. All samples were molded into sheets via hot pressing, and they exhibited significant antibacterial activity (Figure 2). The antibacterial properties of ADIP-PS sheets were evaluated according to ISO 22196:2011, a standard for plastics [33]. Although ISO 22196 typically assesses antibacterial activity against *E. coli* and *S. aureus*, only *S. aureus* was used in this study. Although antibacterial ADIP-PS was synthesized using propan-2-ol in previous studies [25], the ADIP-PS synthesized with toluene also showed high antibacterial activity.

### 3.2. Transparency and Tensile Strength

**Figure 3:**
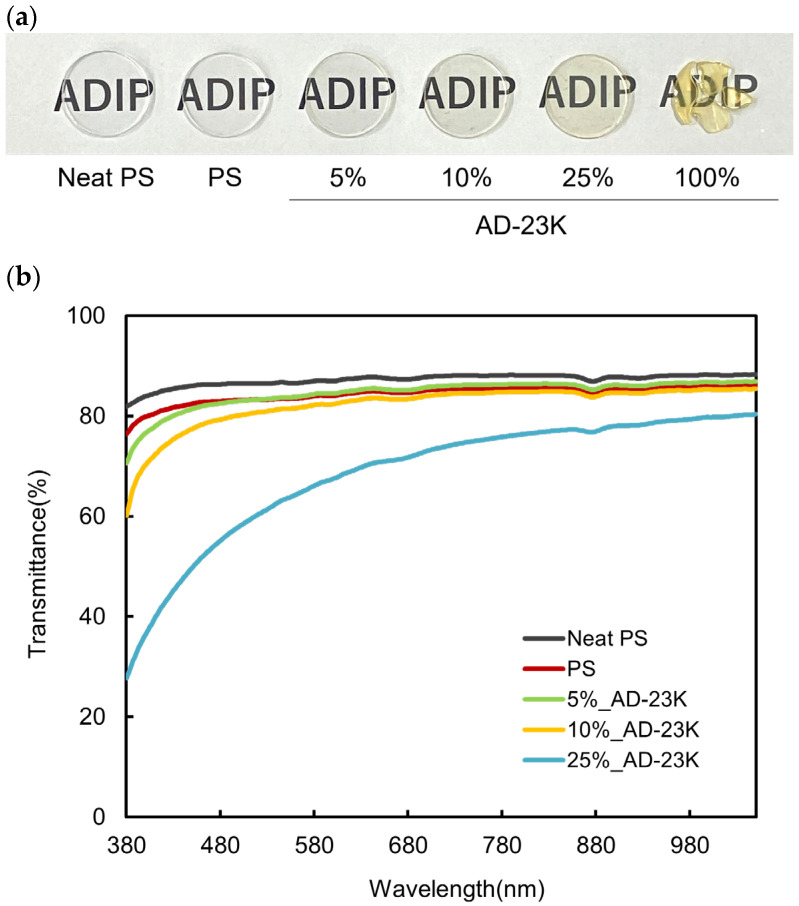
Transparency of ADIP-PS (AD-23K) sheets. (**a**) Representative appearance of PS and ADIP-PS sheets. (**b**) Transmittance spectra of PS and ADIP-PS sheets.

The transparency of ADIP-PS sheets was assessed using a transmittance measurement device. The highest-yield sample, AD-23K, was blended with GPPS at various ratios and molded into sheets. Figure 3a shows the sheets having the thicknesses listed in Table 3. “Neat PS” refers to sheets formed from virgin GPPS pellets, whereas “PS” refers to sheets formed from GPPS pellets after heat blending. Sheets made solely from ADIP-PS (100% AD-23K) were brittle and unsuitable for forming the 2 cm diameter sheets required for transparency evaluation. Other sheets were successfully molded to a uniform thickness of 1.9 mm.

As the ADIP content increased, moderate absorption occurred on the blue side of the visible light region, resulting in a yellow color (Figure 3a). The yellow color is due to the absorption in the UV region (around 250 nm) of imidazolium, as shown by reported absorption spectra [34]. Further, in the 25%_AD-23K samples, absorption occurred across the entire visible light range, and the material became cloudy because of molecular-level insufficient kneading originating from the significant difference in the molecular weights of GPPS and ADIP-PS (Figure 3b). In the 25%_AD-23K samples, absorption across the visible light range and insufficient molecular-level mixing were caused by the significant difference in the molecular weights of GPPS and ADIP-PS, resulting in cloudiness (Figure 3b). Comparing “Neat PS” with “PS”, the heat treatment of GPPS slightly reduced the light absorbance without significantly altering the appearance (Figure 3a), indicating minimal effects from heating. Therefore, the reduction in absorbance observed in the >25% ADIP-PS blends is primarily because of ADIP-induced discoloration and insufficient blending from molecular-weight differences. When the ADIP-PS content was 10% or less, there were no significant changes in appearance compared to “Neat PS” or “PS”, and the light absorbance was unaffected (Figure 3). Additionally, no noticeable changes were observed in the appearance of each sheet even 8 months after molding (Figure S3).

**Figure 4:**
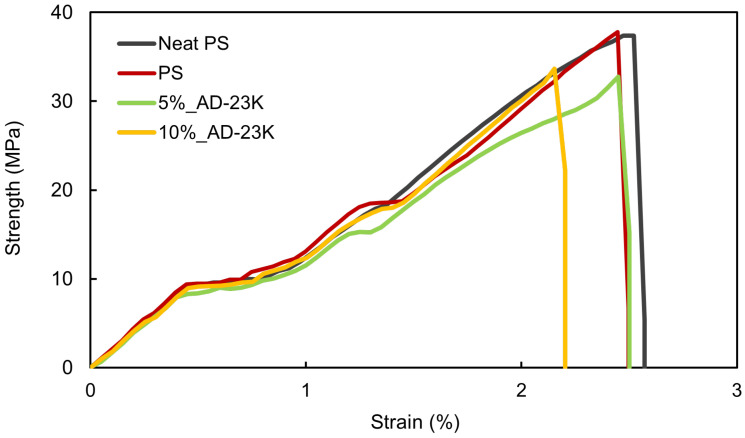
Stress–strain curves of PS and ADIP-PS (AD-23K) resins.

The tensile strength of the PS blended with ADIP-PS was also measured. Both 100%_AD-23K and 25%_AD-23K samples were too brittle for tensile testing because of the lower *M_w_* of AD-23K compared to that of GPPS. Previous studies demonstrated that the factors contributing to the decrease in strength were the reduced entanglement between molecules owing to the smaller molecular weight [35,36] and the decreased interactions between polystyrene molecules because of the hydrophilic functional groups of ADIP [37,38]. Figure 4 shows the stress–strain curves, and the tensile strength, fracture strain, and Young’s modulus values are listed in Table 4. No significant changes in strength were observed between “Neat PS” and “PS” after blending, indicating that blending had no impact on PS strength. The tensile strength and Young’s modulus of 5%_AD-23K and 10%_AD-23K were only slightly lower than those of pure PS, indicating minimal impact on mechanical strength from blending ADIP-PS with GPPS. Based on the transmittance and tensile strength results, blending ADIP-PS with GPPS at ratios of 10% or lower is optimal for maintaining the transparency and mechanical integrity of polystyrene.

### 3.3. Antibacterial Activity of GPPS Blended with ADIP-PS

**Figure 5:**
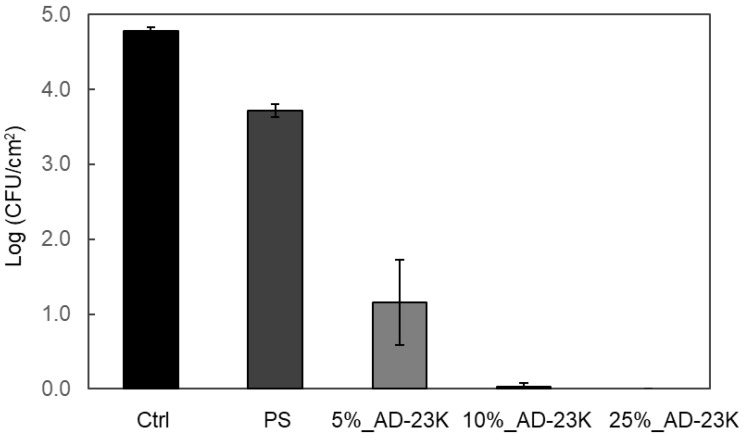
Antibacterial activity of ADIP-PS (AD-23K) sheets with different ratios against *S. aureus*. Error bars represent the standard deviation (SD) with *n* = 3.

The antibacterial activity of GPPS blended with AD-23K is shown in Figure 5. In antibacterial activity evaluation compliant with ISO standards, a reduction of two orders of magnitude in log CFU compared to the control is evaluated as demonstrating significant antibacterial activity. Both 25%_AD-23K and 10%_AD-23K exhibited antibacterial properties similar to those of 100%_AD-23K. In these samples, the log CFU of the *S. aureus* decreased by more than four orders of magnitude compared to the control. Furthermore, in the 5%_AD-23K blend, the log CFU of *S. aureus* decreased by more than three orders of magnitude compared to the control, which is sufficient antibacterial activity. Pure PS showed no significant antibacterial effects. These results suggest that incorporating 5–10% ADIP-PS into GPPS effectively enhances the antibacterial properties without sacrificing transparency or strength (Figure 3 and Figure 4). Figure S4 shows *S. aureus* that was in contact with ADIP-PS kneaded into GPPS for 3 h and stained with PI, a non-membrane permeable fluorescent dye. The staining of *S. aureus* on the ADIP-PS kneaded sheet suggests that the cationic groups on the surface damage the bacterial cell membrane, thereby exhibiting antibacterial properties. By contrast, slight staining by PI was observed on pure PS.

### 3.4. Cationic Groups on the Surface of PS Sheets

**Figure 6:**
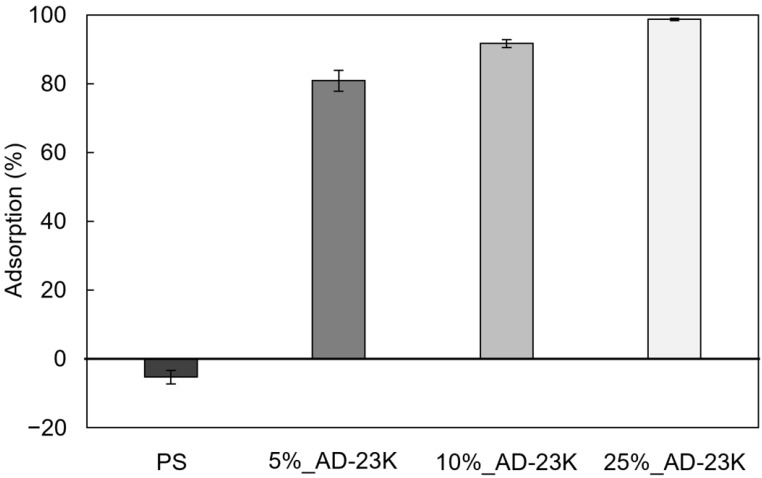
TNS adsorption on GPPS and GPPS blended with ADIP-PS. Error bars represent the SD with *n* = 3.

Cationic surfaces have smaller water contact angles than unmodified surfaces [39]. Therefore, if ADIP-PS imparts antibacterial properties by cationizing the sheet surface, the contact angle decreases. Table 5 shows the contact angles of water on PS sheets with varying ADIP-PS content. The contact angle for PS was approximately 90°. As the ADIP-PS ratio increased, the contact angle decreased. The surface roughness also affects the contact angle [40,41]; thus, we qualitatively evaluated the adsorption of TNS (an anionic fluorescent dye) on the sheet surface. Figure 6 shows the TNS adsorption ratios for GPPS sheets blended with different amounts of ADIP-PS. PS sheets without ADIP-PS showed almost no TNS adsorption. However, as the ADIP-PS ratio increased, TNS adsorption increased correspondingly. This indicates that cationic groups are present on the sheet surface, and their density increases with the increase in ADIP-PS content. Further, the antibacterial activity is correlated with the density of surface cationic groups; thus, blending ADIP-PS with GPPS results in a cationic surface, which results in antibacterial properties.

### 3.5. Comparison of Antibacterial Activity of PS Sheets with Different Molecular Weights

**Figure 7:**
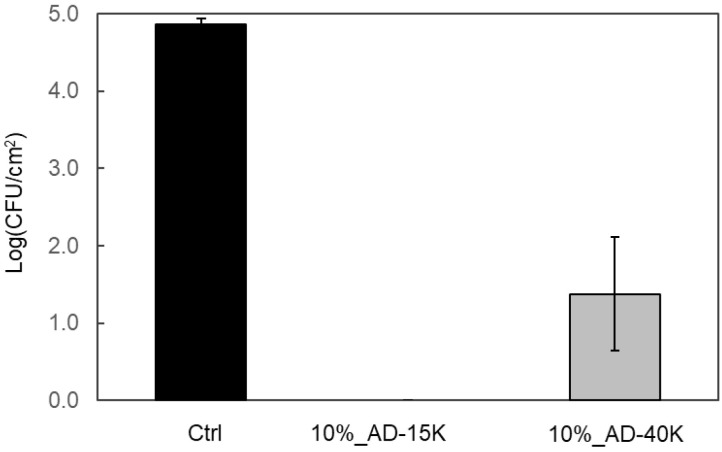
Antibacterial activity of GPPS blended with 10% ADIP-PS sheets. Error bars represent the SD with *n* = 3.

**Figure 8:**
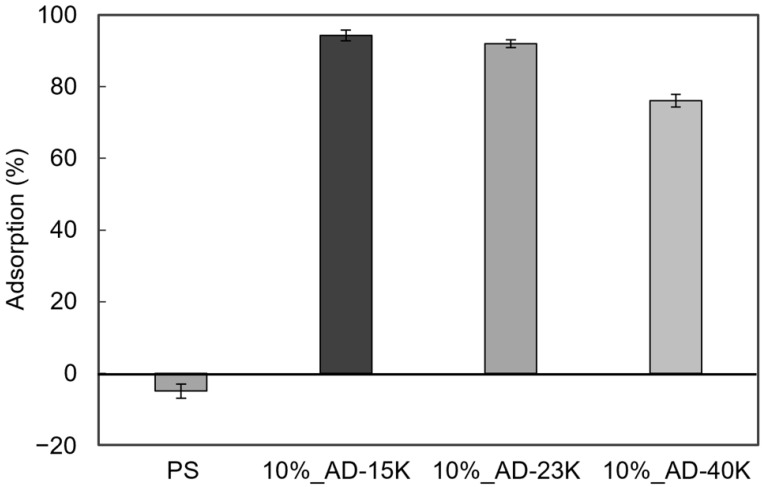
TNS adsorption on PS and GPPS blended with 10% ADIP-PS sheets. Error bars represent the SD with *n* = 3.

Polymer compatibility is affected by chemical structure, molecular weight, polarity, and crystallinity [42]. To evaluate whether ADIP-PS with different molecular weights could maintain antibacterial activity when blended with GPPS, we prepared sheets using 10% blends of AD-15K and AD-40K and assessed their antibacterial properties. AD-15K, which has a lower molecular weight than AD-23K, exhibited similar antibacterial activity to AD-23K, whereas AD-40K, which has a higher molecular weight, also showed strong antibacterial effects (Figure 7). To determine the density of cationic groups on the sheet surfaces, we measured TNS adsorption. AD-15K, which had the highest antibacterial activity, also displayed the highest TNS adsorption rate, whereas AD-40K, with slightly reduced antibacterial activity, showed relatively lower TNS adsorption (Figure 8). This suggests a direct correlation between antibacterial efficacy and cationic group density. Within the tested molecular weight range, blending ADIP-PS with GPPS did not significantly affect antibacterial activity.

## 4. Discussion

A common method for producing antibacterial polystyrene involves mixing the resin with metal nanoparticles, such as silver. However, unlike ADIP-PS, silver can react with hydrogen sulfide in the environment, leading to discoloration and reduced transparency [43]. Additionally, silver-zeolite-containing PS has shown reduced antibacterial effects under wet conditions [25], resulting in long-term performance challenges.

High-molecular-weight antibacterial agents, such as polyhexamethylene biguanide (PHMB) and chitosan, are also commonly used [44,45,46]. However, adding PHMB to polyurethane at concentrations as low as 5% significantly decreases the tensile strength of polyurethane fibers [44]. Similarly, chitosan incorporation into polylactic acid (PLA) results in substantial strength loss because of solubility issues [47] resulting from the incompatibility of chemical structures and polarity differences between the two polymers. Consequently, the interactions between the base polymer and additives are affected. In the case of chitosan and PLA, blending caused discontinuities in the PLA matrix because of poor solubility [47]. Using the same polymer as an antibacterial additive could resolve such challenges but is not straightforward. Nevertheless, this study demonstrates the effectiveness of using ADIP-PS, a contact-killing antibacterial agent synthesized with a cationic polymerization initiator. Typically, lower-molecular-weight polymers are more likely to be exposed on the surface when blended with the same polymer type [48,49], providing a potential advantage for ADIP-PS in maintaining antibacterial functionality.

Theoretical analysis suggests several reasons for the tendency of low-molecular-weight polymers to be positioned more easily on the surface. These include entropy-driven behavior, shorter chains being closer to the surface, and fewer possible configurations compared to longer chains, which lowers the entropy penalty [50,51]. As shown in Figure 7, the 10% AD-40K blend, which has a higher molecular weight, showed slightly reduced antibacterial activity. Additionally, Figure 8 shows a lower level of exposed cationic groups. However, this does not mean that a lower molecular weight always improves the antibacterial properties. Previous studies have reported a significant decrease in the glass transition temperature (*T_g_*) of linear PS when the *M_n_* falls below 18,000 [52,53]. Blending polymers with significantly different *T_g_* values can affect the bulk resin properties, making extremely low-molecular-weight ADIP-PS unsuitable as an antibacterial agent. Based on these factors, AD-23K (*M_n_* = 23,000) was considered within the optimal molecular weight range. We also investigated the surface segregation potential because of the presence of cationic groups at the ends of ADIP-PS chains. Our results demonstrate that even with a 5% blend and a molecular weight of approximately 23,000, ADIP-PS shows sufficient antibacterial activity. Notably, each ADIP-PS chain contained at most two cationic groups, representing an extremely small weight percentage. In the 5% ADIP-PS blend, cationic groups comprised only about 0.1% by weight, significantly lower than high-molecular-weight cationic agents such as PHMB [44] and chitosan [47]. Previous studies on polymer end-group chemistry have shown that PS with hydrophilic end-groups tends to undergo internal segmentation, resulting in the accumulation of hydrophilic groups on the surface [54]. Given the hydrophilic nature of the imidazolium groups used in our study, a similar effect likely occurred.

Surface segregation is more common in low-molecular-weight polymers. In this study, AD-15K, the lowest molecular weight polymer, had the highest concentration of cationic groups on its surface (Figure 8). However, the effect of molecular weight on surface segregation depends on the interface. For example, low-molecular-weight polymers are more likely to concentrate at air interfaces, whereas higher-molecular-weight polymers tend to concentrate at solid interfaces [55]. The surface properties of materials also vary depending on the manufacturing method, presenting additional opportunities for research.

There are two main challenges to commercializing ADIP-PS as an antimicrobial additive. First, its application in continuous PS product production. Many packaging products, such as food containers, are manufactured via extrusion molding. For ADIP-PS to be effective, the cationic groups must be present on the resin surface. Further studies are required to assess whether small amounts of ADIP-PS can maintain antibacterial activity under typical commercial production methods. Second, extending its application to other styrene-based resins, such as acrylonitrile–butadiene–styrene and styrene–acrylonitrile (SAN), could significantly expand its potential uses. Future research could explore polymer synthesis using ADIP and optimization of blending conditions. This study serves as a foundation for addressing these challenges.

## 5. Conclusions

This paper presents a novel method to impart antibacterial properties to PS through thermal blending while preserving its transparency and strength. Previously, we have shown that antibacterial PS can be synthesized using the cationic radical polymerization initiator ADIP. However, the synthesized ADIP-PS had low material strength and exhibited yellow discoloration, limiting practical applications [25]. In this study, we demonstrated the potential of ADIP-PS as an antibacterial additive. ADIP-PS, having *M_n_* values between 15,000 and 40,000, was optimal for achieving that purpose. By thermally blending 5–10 wt% ADIP-PS with GPPS, antibacterial properties were successfully imparted while maintaining the material’s original properties. The blended resin surface exhibited cationic groups and was confirmed to be hydrophilic.

## Figures and Tables

**Table 1:** Polymerization reactions of PS with ADIP as an initiator.

Sample	Reaction Temperature (°C)	Reaction Time (h)
AD-15K	40	24
AD-23K	40	48
AD-40K	60	24

**Table 2:** Yield, molecular weight, and ADIP residue contents of the obtained ADIP-PS.

Sample	*M_n_* ^a^	*M_w_* ^a^	*M_w_*/*M_n_*	Yield (%)	N Content (wt%)	ADIP Residue Content (mol%)
AD-15K	15,000	54,000	3.6	40	0.83	4.3
AD-23K	23,000	120,000	5.3	56	0.66	3.4
AD-40K	40,000	310,000	7.7	33	0.55	2.8

^a^ *M_n_* and *M_w_* evaluated by GPC.

**Table 3:** Thickness of PS and ADIP-PS (AD-23K) sheet used for transparency evaluation.

Sample	Thickness (mm)
Neat PS	1.92
PS	1.91
5%_AD-23K	1.89
10%_AD-23K	1.89
25%_AD-23K	1.86
100%_AD-23K	Not formed

**Table 4:** Mechanical properties of PS and ADIP-PS (AD-23K) resins.

Sample	Tensile Strength (MPa)	Elongation at Break (%)	Young’s Modulus (MPa)
Neat PS	37.4	2.6	2108
PS	37.8	2.5	2100
5%_AD-23K	32.8	2.5	1979
10%_AD-23K	33.7	2.2	1886

**Table 5:** Contact angles of GPPS sheets and GPPS blended with ADIP-PS.

Sample	Contact Angle (°)
PS	88 ± 0.8
5%_AD-23K	73 ± 1.5
10%_AD-23K	66 ± 5.0
25%_AD-23K	63 ± 1.2

## Data Availability

Data will be made available on request.

## Supplementary Materials

The following supporting information can be downloaded at: https://www.mdpi.com/article/10.3390/polym16223167/s1, Figure S1: FTIR spectra of ADIP-PSs with different molecular weights; Figure S2: FTIR spectra of ADIP-PS (AD-23K, green) and heat-treated ADIP-PS (AD-23K, red); Figure S3: Transparency of ADIP-PS (AD-23K) sheets after 8 months; Figure S4: Representative images of *S. aureus* on a PS and 10%_AD-23K sheets were recorded by laser scanning confocal fluorescence microscopy. PI: Ex. 561 nm, Em. 565–727 nm, SYTO 9: Ex. 488 nm, Em. 380–548 nm. The white scale bar indicates 5 μm.
