# Supplementary material for: Antibacterial, Transparency, and Mechanical Properties of Cationic Radical Initiator Triggered Polystyrene Sheets Obtained by Thermal Blending
Source: Polymers (Basel). 2024 Nov 13;16(22):3167. doi: 10.3390/polym16223167 (PMC11598219; doi:10.3390/polym16223167)
Supplement: Supplementary file 1 [file polymers-16-03167-s001.zip › polymers-3261159-supplementary.pdf]

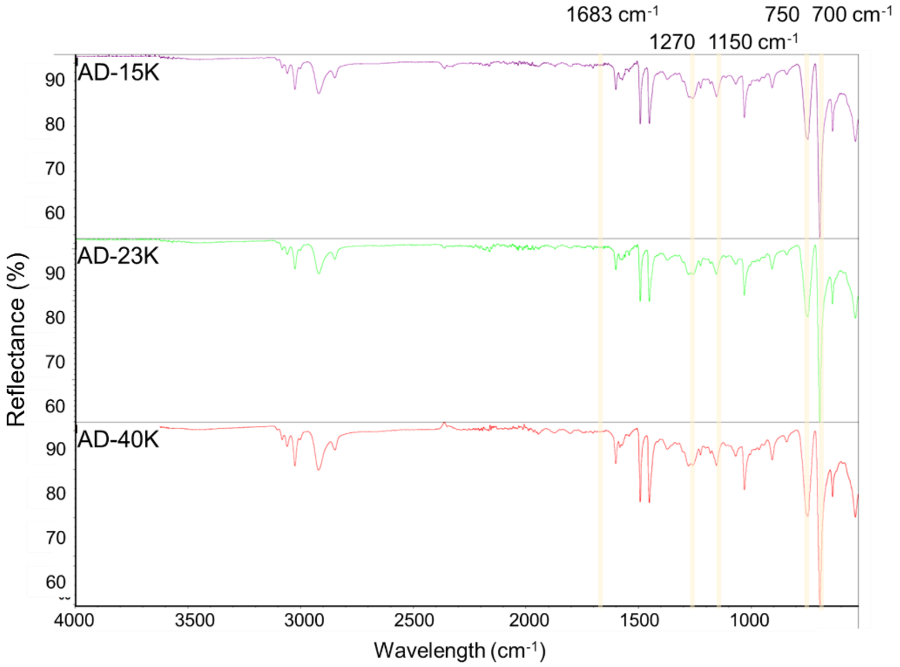

Figure S1. FTIR spectra of ADIP-PSs with different molecular weights.

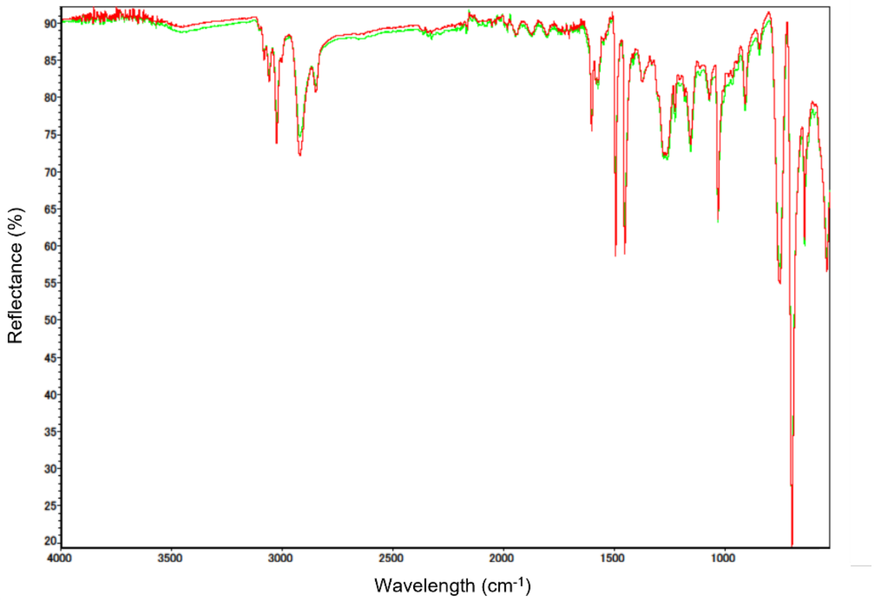

Figure S2. FTIR spectra of ADIP-PS (AD-23K, green) and heat-treated ADIP-PS (AD-23K, red).

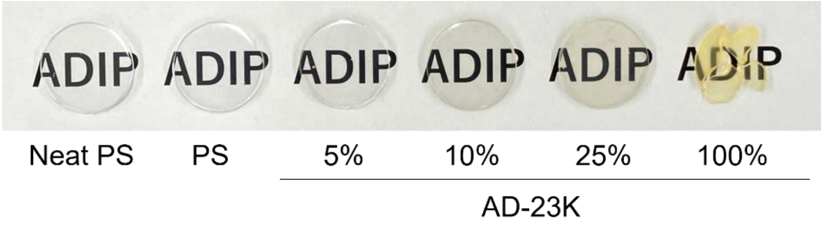

Figure S3. Transparency of ADIP-PS (AD-23K) sheets after 8 months.

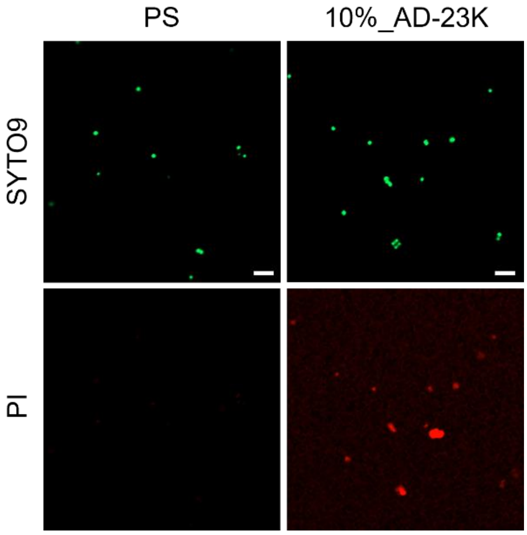

Figure S4. Representative images of *S. aureus* on PS and 10%_AD-23K sheets were recorded by laser scanning confocal fluorescence microscopy. PI: Ex. 561 nm, Em. 565–727 nm, SYTO 9: Ex. 488 nm, Em. 380–548 nm. The white scale bar indicates 5 μm.
